# Spt6: Two fundamentally distinct functions in the regulation of histone modification

**DOI:** 10.4161/epi.26487

**Published:** 2013-10-09

**Authors:** Hiroaki Kato, Kosuke Okazaki, Takeshi Urano

**Affiliations:** 1 Department of Biochemistry; Shimane University School of Medicine; Izumo, Japan; 2 PRESTO; Japan Science and Technology Agency (JST); Saitama, Japan

**Keywords:** Spt6, epigenetics, RNA polymerase II, posttranslational modifications, histone methylation

## Abstract

As posttranslational modifications of histones H3 and H4 determine the state of chromatin in cis, these histones should remain attached to template DNA during transcription in order to maintain the state of chromatin. RNA polymerase II itself can transcribe the nucleosome template without changing nucleosome positioning. However, it was uncertain whether Spt6, a highly conserved polymerase-associated histone chaperone, prevents “preexisting” histone molecules from being dissociated from template DNA during transcription. We recently showed that Spt6 prevents transcription-coupled loss of posttranslationally modified histone H3. Taking previous studies into account, we would like to propose here that Spt6 has two fundamentally distinct functions in the regulation of histone modification: one is to act as a platform for histone modifiers and the other is to act as a molecular liaison between histone molecules and template DNA to prevent cotranscriptional dissociation of preexisting histones in order to maintain locus-specific modifications.

## Introduction

Positioning and posttranslational modifications of histones determine the state of chromatin in cis.^1^^,^^2^ Of the three canonical RNA polymerases in eukaryotes, only RNA polymerase II (RNAP II) can transcribe nucleosome templates without changing nucleosome positioning in vitro.^3^ Genomic regions transcribed by RNAP II, with the exception of transcription start and termination sites, are generally filled with nucleosome arrays.^4^^-^^7^ As the formation of small, intranucleosomal loops (“zero-size” loops) ensures transcriptional elongation through the nucleosome, histones H3 and H4 need not to be fully dissociated from the template DNA.^8^ Paradoxically, it is also known that transcription by RNAP II per se has the potential to dissociate histones H3 and H4 from the template,^9^^,^^10^ suggesting the existence of factors that prevent cotranscriptional histone dissociation in order to maintain epigenetic integrity.

The tight relationship between RNAP II and histones implies that coincidental emergence of these molecules would have been an important step in the acquisition of the histone-based layer of epigenetic regulation. Another important molecule in this respect would have been Spt6 (Suppressor of Ty 6), a highly conserved protein that is recognized as both a transcription elongation factor^11^^-^^14^ and a chaperone for histones H3 and H4.^13^^,^^15^ Spt6 has three functionally distinct regions: the N-terminus, a Tex-like core, and the C-terminus.^16^^,^^17^ The Tex-like core region is conserved across the biological domains bacteria, archaea, and eukarya. In contrast, the N- and C-terminal regions are conserved only in the eukarya. The N-terminal region is essential for the histone chaperone activity of Spt6.^15^^,^^18^ The C-terminal region has a particular domain called tandem Src homology 2 (tSH2), which interacts directly with phosphorylated forms of the C-terminal domain (CTD) of Rpb1, the largest subunit of RNAP II (RNAP II CTD).^19^ Spt6 is recruited to transcribed regions as a component of the general RNAP II transcription complex, mainly through interaction between its tSH2 domain and the RNAP II CTD.^20^ Here, we would like to discuss two fundamentally distinct functions of Spt6 in epigenetic regulation: (1) recruitment of histone modifiers to target genes, and (2) prevention of transcription-coupled loss of preexisting posttranslationally modified histone molecules (Fig. 1).

**Figure:**
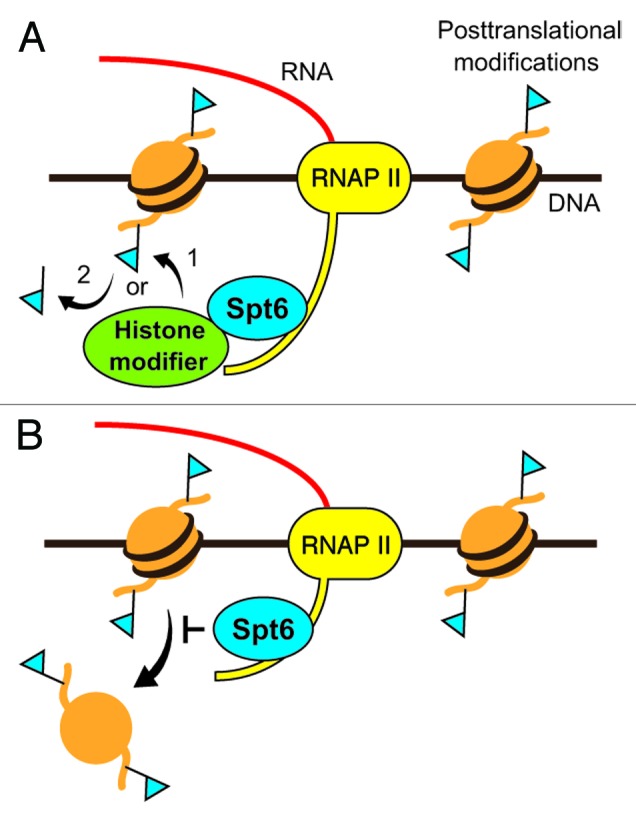
**Figure 1.** Two fundamentally distinct functions of Spt6 in the regulation of histone modification. (**A**) Spt6 serves as a transcription machinery-anchored platform for the recruitment of histone modifiers to target loci. Budding yeast Spt6 assists Set2, which interacts with the RNAP II CTD, to trimethylate histone H3 at Lys-36 during transcription (see arrow #1). In mammals, SETD2 is recruited to target loci through interaction with Iws1, a highly conserved Spt6-interacting protein. For demethylation of histone H3, Spt6 recruits JMJD3/KDM6B, KDM6A, and KIAA1718 to target loci (see arrow #2). (**B**) Spt6 serves as a molecular liaison that prevents cotranscriptional dissociation of preexisting histones H3 and H4. At a minimum, this function is required for the maintenance of histone H3 methylation at Lys-4 and Lys-9 in euchromatin and heterochromatin, respectively. Other posttranslational modifications of histone molecules could also be maintained through this function.

## Positive Regulation by Spt6 of Histone H3 Trimethylation at Lys-36

The tail of histone H3 is marked with various posttranslational modifications, including acetylations and methylations.^1^^,^^2^ Methylation of histone H3 at Lys-36 is usually observed in transcribed regions of the genome and is important for repressing the histone exchange that leads to impaired histone acetylation and aberrant transcription.^21^^-^^24^ In the budding yeast *Saccharomyces cerevisiae*, the *spt6–1004* mutant allele, which encodes an Spt6 protein lacking the helix-hairpin-helix domain within the Tex-like core, causes a loss of histone H3 trimethylation at Lys-36 (H3K36me3) that cannot be suppressed by exogenous expression of Set2,^25^ a Lys-36–specific methyltransferase that interacts directly with elongating RNAP II.^26^ Spt6 also influences Set2-dependent positioning of H3K36me3 in budding yeast.^25^

The Spt6-mediated regulation of H3K36me3 is not restricted to budding yeast but is also found in mammals. Yoh et al. showed that the Set2 homolog SETD2 is recruited to specific target genes such as *PABPC1* through interaction between SETD2 and Iws1, an Spt6-interacting protein.^27^ Begum et al. reported that in the mouse B-cell lymphoma line CH12F3–2A, Spt6, which activates class-switch recombination at the immunoglobulin heavy chain locus, positively regulates the levels of H3K36me3 and trimethylation of histone H3 at Lys-4 (H3K4me3, discussed below).^28^ The evidence from these studies has clarified the role of Spt6 in the positive regulation of H3K36me3 in transcribed regions.

## Negative Regulation by Spt6 of Histone H3 Trimethylation at Lys-27

In contrast, recent studies revealed the involvement of Spt6 in negative regulation of trimethylation of histone H3 at Lys-27 (H3K27me3), a mark of epigenetically repressed genes.^29^ Chen et al. identified Spt6 as a component of the JMJD3 (also known as KDM6B) complex.^30^ JMJD3 is a Lys-27–specific histone demethylase that plays important roles in transcriptional regulation and cell differentiation.^31^^,^^32^ The JMJD3 complex also includes SETD2 and another histone demethylase, KIAA1718.^30^ In HL-60 human promyelocytic leukemia cells, demethylation of H3K27me3 and an increase in Spt6 occupancy specifically occurs in differentiation-induced genes (such as *CXCL3*) in a JMJD3- and KIAA1718-dependent manner. Therefore, these histone demethylases appear to positively regulate Spt6 localization and transcriptional elongation for effective demethylation of H3K27me3 in order to activate differentiation-induced genes.

Wang et al. also reported on the relationship between differentiation-induced H3K27me3 demethylation and Spt6.^33^ They showed that Spt6 interacts with the JMJD3 paralogue KDM6A and is necessary for skeletal muscle cell differentiation. Knockdown of Spt6 in differentiated C2C12 skeletal myogenic cells results in an error in differentiation-induced demethylation of H3K27me3 at the target genes, which is partially suppressed by additional knockdown of Ezh2, the H3K27me3-specific methyltransferase component of the PRC2 complex.^29^ Spt6-dependent H3K27me3 demethylation has also been observed in zebrafish.^33^ Together, these data indicate that in vertebrates Spt6 recruits histone demethylases to target genes to induce demethylation of H3K27me3 for transcriptional activation.

## Spt6 Prevents Transcription-Coupled Loss of Preexisting Histone H3

The observation that the *spt6–1004* mutation in budding yeast causes loss of the nucleosome at highly transcribed genes^34^ led to the well-accepted idea that, as an elongating RNAP II-attached histone chaperone, Spt6 reassembles/restores nucleosomes following passage of RNAP II at highly transcribed regions. Reassembly of nucleosomes by Spt6 could be performed either with free histone molecules that are reserved in the nucleoplasm or with the preexisting histone molecules on the template. The positional stability of histone H3 and H4 molecules against transcription^35^^-^^37^ had suggested that local preexisting histone molecules are reused for nucleosome reassembly. However, there was no evidence showing that Spt6 prevents loss of preexisting histone molecules. In addition, it was uncertain whether a complete deletion of the Spt6 gene affects nucleosome occupancy only at highly transcribed genes or also at rarely transcribed genes. Furthermore, as the *spt6–1004* mutation has no effect on global levels of di- or tri-methylation of histone H3 at Lys-4 (H3K4me2 and H3K4me3, respectively),^38^ this mutation does not appear to be useful for evaluating the relationship between Spt6 and histone H3 methylation at Lys-4.

Kielly et al. reported that in the fission yeast *Schizosaccharomyces pombe*, a mutation designated *spt6–1* that mimics the budding yeast *spt6–1004* mutation causes a decrease in the level of histone H3 Lys-9 trimethylation at heterochromatic regions.^39^Similar to the case in budding yeast, the fission yeast *spt6–1* mutation causes nucleosome loss at highly transcribed euchromatic regions. However, this mutation does not significantly impact nucleosome positioning and the level of histone H3 Lys-9 dimethylation (H3K9me2) in heterochromatin. Although Spt6 has long been regarded as essential for cell viability, fission yeast cells are able to grow without expressing this protein, although growth of Spt6 deletant cells is extremely slow.^39^

In addition to the above-mentioned phenotypes caused by partial Spt6 inactivation, we recently found that complete deletion of *spt6* leads to loss of H3K9me2 in heterochromatin as well as loss of H3K4me2 and H3K4me3 in euchromatin.^40^ The loss rates for these posttranslational marks and the incorporation rate of Lys-56–acetylated histone H3, which does not carry locus-specific posttranslational modifications,^23^ apparently correlate with the rate of cotranscriptional nucleosome loss. In addition, in the absence of Spt6, a significant reduction in histone H3 occupancy is detectable even at rarely expressed genes and is accompanied by impaired transcriptional repression. These observations indicate that Spt6 prevents transcription-coupled loss of preexisting histones H3 and H4 in order to maintain the state of locus-specific histone modification.

There is some evidence confirming Spt6-dependent regulation of H3K4me3. Wang et al. showed that a decrease in H3K4me3 is observed only in the myogenin gene, which exhibited the highest extent of transcriptional induction among the genes tested.^33^It is possible that partial inactivation of Spt6 causes a cotranscriptional dissociation of Lys-4–methylated histone H3 that can only be observed in highly transcribed genes. Begum et al. proposed that regulation of H3K4me3 through Spt6 is required for determining the targets of activation-induced cytidine deaminase.^28^ Through co-immunoprecipitation analyses, they showed that formation of a complex between Spt6 and Set1A, a mammalian homolog of Set1, requires the tSH2 domain of Spt6, suggesting that Spt6 recruits Set1A to the targets during transcription. Set1A complex is known to interact directly with RNAP II.^41^ Additionally, the tSH2 domain is required for interaction between Spt6 and RNAP II.^19^ Thus, it is also possible that an indirect interaction between Spt6 and Set1A through RNAP II is compromised by deletion of tSH2, and that the decrease in the level of H3K4me3 caused by Spt6 knockdown could also be a result of the observed loss of histone H3.^28^

## Perspectives

Based on the currently available evidence, Spt6 appears to have two fundamentally distinct functions in the regulation of histone modification. One function is to serve as a transcription machinery-anchored platform for recruitment of histone modifiers to target loci in order to enhance their functions (Fig. 1A). This function of Spt6 is required for changing the state of chromatin by writing or erasing modifications and is also required for maintaining the chromatin state through continuous writing of modifications against antagonistic erasing activities. Some questions arise regarding this function. For instance, what determines the target loci of histone modifiers when Spt6 is recruited to the transcribed region as a component of the general RNAP II transcription complex? Also, how does the first transcription occur at repressed loci to increase the transcription level (e.g., through demethylation of H3K27me3)?

We would like to stress that the other fundamental function of Spt6 is to serve as a molecular liaison between histone molecules and DNA in order to prevent cotranscriptional dissociation of preexisting histones H3 and H4 (Fig. 1B). It is now clear that this function is not restricted to highly transcribed genes. Through this function, Spt6 plays a critical role in the maintenance of locus-specific histone modifications. In other words, without this histone chaperone, even a single sweep of transcription could result in loss of cognate epigenetic information. Given the fact that almost all of the genome is transcribed to some extent,^42^ insufficient Spt6 activity may lead to a catastrophic breakdown in epigenomic integrity. The emergence in eukaryotes of the particular form of this protein with histone chaperone and RNAP II-interacting regions suggests that Spt6 may function in a triadic manner with histone molecules and RNAP II as the “ridges” expressed on the epigenetic landscape.

Preexisting locus-specific epigenetic information could be erased if the solidarity between RNAP II, histones, and Spt6 is actively hindered. For example, the RNAP II activity that triggers alteration of the chromatin state of previously repressed promoters^43^^-^^45^could be accompanied by lower Spt6 activity. In this regard, the fact that Iws1 physically interacts with Spt6 to prevent its binding to the nucleosome is very suggestive.^18^ To date, no other candidate negative regulators of the histone chaperone activity of Spt6 have been reported. It also remains unclear whether the functions of Spt6 and Iws1 are themselves regulated through posttranslational modification. Furthermore, the impact of Spt6 breakdown on the accumulation of epigenetic abnormalities in cancer cells has yet to be evaluated. Therefore, in order to more fully understand the effects of the cotranscriptional epigenetic regulation and epigenetic disorder that are frequently observed in cancer cells,^46^ the various aspects of the functions of Spt6 discussed here should be studied further at the molecular level.

## Notes

KatoHOkazakiKIidaTNakayamaJMurakamiYUranoT Spt6 prevents transcription-coupled loss of posttranslationally modified histone H3 Sci Rep 2013 3 2186 10.1038/srep02186

## Abbreviations:

CTD: C-terminal domain
H3K4me2: histone H3 dimethylation at Lys-4
H3K4me3: histone H3 trimethylation at Lys-4
H3K9me2: histone H3 dimethylation at Lys-9
H3K27me3: histone H3 trimethylation at Lys-27
H3K36me3: histone H3 trimethylation at Lys-36
RNAP II: RNA polymerase II
Spt6: Suppressor of Ty 6
tSH2: tandem Src homology 2

## Notes

10.4161/epi.26487

## References

[1] Zentner GE, Henikoff S (2013). Regulation of nucleosome dynamics by histone modifications. Nat Struct Mol Biol.

[2] Smolle M, Workman JL (2013). Transcription-associated histone modifications and cryptic transcription. Biochim Biophys Acta.

[3] Kireeva ML, Walter W, Tchernajenko V, Bondarenko V, Kashlev M, Studitsky VM (2002). Nucleosome remodeling induced by RNA polymerase II: loss of the H2A/H2B dimer during transcription. Mol Cell.

[4] Lee W, Tillo D, Bray N, Morse RH, Davis RW, Hughes TR, Nislow C (2007). A high-resolution atlas of nucleosome occupancy in yeast. Nat Genet.

[5] Mavrich TN, Jiang C, Ioshikhes IP, Li X, Venters BJ, Zanton SJ, Tomsho LP, Qi J, Glaser RL, Schuster SC (2008). Nucleosome organization in the Drosophila genome. Nature.

[6] Mavrich TN, Ioshikhes IP, Venters BJ, Jiang C, Tomsho LP, Qi J, Schuster SC, Albert I, Pugh BF (2008). A barrier nucleosome model for statistical positioning of nucleosomes throughout the yeast genome. Genome Res.

[7] Wilhelm BT, Marguerat S, Aligianni S, Codlin S, Watt S, Bähler J (2011). Differential patterns of intronic and exonic DNA regions with respect to RNA polymerase II occupancy, nucleosome density and H3K36me3 marking in fission yeast. Genome Biol.

[8] Kulaeva OI, Gaykalova DA, Pestov NA, Golovastov VV, Vassylyev DG, Artsimovitch I, Studitsky VM (2009). Mechanism of chromatin remodeling and recovery during passage of RNA polymerase II. Nat Struct Mol Biol.

[9] Kulaeva OI, Hsieh FK, Studitsky VM (2010). RNA polymerase complexes cooperate to relieve the nucleosomal barrier and evict histones. Proc Natl Acad Sci U S A.

[10] Lee CK, Shibata Y, Rao B, Strahl BD, Lieb JD (2004). Evidence for nucleosome depletion at active regulatory regions genome-wide. Nat Genet.

[11] Endoh M, Zhu W, Hasegawa J, Watanabe H, Kim DK, Aida M, Inukai N, Narita T, Yamada T, Furuya A (2004). Human Spt6 stimulates transcription elongation by RNA polymerase II in vitro. Mol Cell Biol.

[12] Ardehali MB, Yao J, Adelman K, Fuda NJ, Petesch SJ, Webb WW, Lis JT (2009). Spt6 enhances the elongation rate of RNA polymerase II in vivo. EMBO J.

[13] Duina AA (2011). Histone Chaperones Spt6 and FACT: Similarities and Differences in Modes of Action at Transcribed Genes. Genet Res Int.

[14] Hartzog GA, Wada T, Handa H, Winston F (1998). Evidence that Spt4, Spt5, and Spt6 control transcription elongation by RNA polymerase II in Saccharomyces cerevisiae. Genes Dev.

[15] Bortvin A, Winston F (1996). Evidence that Spt6p controls chromatin structure by a direct interaction with histones. Science.

[16] Close D, Johnson SJ, Sdano MA, McDonald SM, Robinson H, Formosa T, Hill CP (2011). Crystal structures of the S. cerevisiae Spt6 core and C-terminal tandem SH2 domain. J Mol Biol.

[17] Johnson SJ, Close D, Robinson H, Vallet-Gely I, Dove SL, Hill CP (2008). Crystal structure and RNA binding of the Tex protein from Pseudomonas aeruginosa. J Mol Biol.

[18] McDonald SM, Close D, Xin H, Formosa T, Hill CP (2010). Structure and biological importance of the Spn1-Spt6 interaction, and its regulatory role in nucleosome binding. Mol Cell.

[19] Mayer A, Heidemann M, Lidschreiber M, Schreieck A, Sun M, Hintermair C, Kremmer E, Eick D, Cramer P (2012). CTD tyrosine phosphorylation impairs termination factor recruitment to RNA polymerase II. Science.

[20] Mayer A, Lidschreiber M, Siebert M, Leike K, Söding J, Cramer P (2010). Uniform transitions of the general RNA polymerase II transcription complex. Nat Struct Mol Biol.

[21] Smolle M, Workman JL, Venkatesh S (2013). reSETting chromatin during transcription elongation. Epigenetics.

[22] Smolle M, Venkatesh S, Gogol MM, Li H, Zhang Y, Florens L, Washburn MP, Workman JL (2012). Chromatin remodelers Isw1 and Chd1 maintain chromatin structure during transcription by preventing histone exchange. Nat Struct Mol Biol.

[23] Venkatesh S, Smolle M, Li H, Gogol MM, Saint M, Kumar S, Natarajan K, Workman JL (2012). Set2 methylation of histone H3 lysine 36 suppresses histone exchange on transcribed genes. Nature.

[24] Maltby VE, Martin BJ, Schulze JM, Johnson I, Hentrich T, Sharma A, Kobor MS, Howe L (2012). Histone H3 lysine 36 methylation targets the Isw1b remodeling complex to chromatin. Mol Cell Biol.

[25] Youdell ML, Kizer KO, Kisseleva-Romanova E, Fuchs SM, Duro E, Strahl BD, Mellor J (2008). Roles for Ctk1 and Spt6 in regulating the different methylation states of histone H3 lysine 36. Mol Cell Biol.

[26] Vojnic E, Simon B, Strahl BD, Sattler M, Cramer P (2006). Structure and carboxyl-terminal domain (CTD) binding of the Set2 SRI domain that couples histone H3 Lys36 methylation to transcription. J Biol Chem.

[27] Yoh SM, Lucas JS, Jones KA (2008). The Iws1:Spt6:CTD complex controls cotranscriptional mRNA biosynthesis and HYPB/Setd2-mediated histone H3K36 methylation. Genes Dev.

[28] Begum NA, Stanlie A, Nakata M, Akiyama H, Honjo T (2012). The histone chaperone Spt6 is required for activation-induced cytidine deaminase target determination through H3K4me3 regulation. J Biol Chem.

[29] Cao R, Zhang Y (2004). The functions of E(Z)/EZH2-mediated methylation of lysine 27 in histone H3. Curr Opin Genet Dev.

[30] Chen S, Ma J, Wu F, Xiong LJ, Ma H, Xu W, Lv R, Li X, Villen J, Gygi SP (2012). The histone H3 Lys 27 demethylase JMJD3 regulates gene expression by impacting transcriptional elongation. Genes Dev.

[31] Burgold T, Spreafico F, De Santa F, Totaro MG, Prosperini E, Natoli G, Testa G (2008). The histone H3 lysine 27-specific demethylase Jmjd3 is required for neural commitment. PLoS One.

[32] Agger K, Cloos PA, Christensen J, Pasini D, Rose S, Rappsilber J, Issaeva I, Canaani E, Salcini AE, Helin K (2007). UTX and JMJD3 are histone H3K27 demethylases involved in HOX gene regulation and development. Nature.

[33] Wang AH, Zare H, Mousavi K, Wang C, Moravec CE, Sirotkin HI, Ge K, Gutierrez-Cruz G, Sartorelli V (2013). The histone chaperone Spt6 coordinates histone H3K27 demethylation and myogenesis. EMBO J.

[34] Ivanovska I, Jacques PE, Rando OJ, Robert F, Winston F (2011). Control of chromatin structure by spt6: different consequences in coding and regulatory regions. Mol Cell Biol.

[35] Dion MF, Kaplan T, Kim M, Buratowski S, Friedman N, Rando OJ (2007). Dynamics of replication-independent histone turnover in budding yeast. Science.

[36] Schwartz BE, Ahmad K (2005). Transcriptional activation triggers deposition and removal of the histone variant H3.3. Genes Dev.

[37] Thiriet C, Hayes JJ (2005). Replication-independent core histone dynamics at transcriptionally active loci in vivo. Genes Dev.

[38] Kaplan CD, Holland MJ, Winston F (2005). Interaction between transcription elongation factors and mRNA 3′-end formation at the Saccharomyces cerevisiae GAL10-GAL7 locus. J Biol Chem.

[39] Kiely CM, Marguerat S, Garcia JF, Madhani HD, Bähler J, Winston F (2011). Spt6 is required for heterochromatic silencing in the fission yeast Schizosaccharomyces pombe. Mol Cell Biol.

[40] Kato H, Okazaki K, Iida T, Nakayama J, Murakami Y, Urano T (2013). Spt6 prevents transcription-coupled loss of posttranslationally modified histone H3. Sci Rep.

[41] Lee JH, Skalnik DG (2008). Wdr82 is a C-terminal domain-binding protein that recruits the Setd1A Histone H3-Lys4 methyltransferase complex to transcription start sites of transcribed human genes. Mol Cell Biol.

[42] Wilhelm BT, Marguerat S, Watt S, Schubert F, Wood V, Goodhead I, Penkett CJ, Rogers J, Bähler J (2008). Dynamic repertoire of a eukaryotic transcriptome surveyed at single-nucleotide resolution. Nature.

[43] Orphanides G, Reinberg D (2000). RNA polymerase II elongation through chromatin. Nature.

[44] Hirota K, Ohta K (2009). Transcription of mRNA-type long non-coding RNAs (mlonRNAs) disrupts chromatin array. Commun Integr Biol.

[45] Hirota K, Miyoshi T, Kugou K, Hoffman CS, Shibata T, Ohta K (2008). Stepwise chromatin remodelling by a cascade of transcription initiation of non-coding RNAs. Nature.

[46] Berdasco M, Esteller M (2010). Aberrant epigenetic landscape in cancer: how cellular identity goes awry. Dev Cell.

